# Does health-related quality of life among adults with pulmonary tuberculosis improve across the treatment period? A hospital-based cross sectional study in Mbale Region, Eastern Uganda

**DOI:** 10.1186/s13104-016-2277-y

**Published:** 2016-10-18

**Authors:** Stevens M. B. Kisaka, Elizeus Rutebemberwa, Simon Kasasa, Francis Ocen, Joan Nankya-Mutyoba

**Affiliations:** 1 Department of Epidemiology and Biostatistics, Makerere University School of Public Health, P.O.BOX 7072, Kampala, Uganda; 2 Department of Health Policy, Planning and ManagementMakerere University School of Public Health, Kampala, Uganda; 3 College of Veterinary Medicine Animal Resources and Biosecurity, Makerere University, Kampala, Uganda

**Keywords:** Tuberculosis, Health-related quality of life, Smear-positive pulmonary tuberculosis, SF-36v2 health survey form, Uganda

## Abstract

**Background:**

Most tuberculosis (TB) case management guidelines emphasize microbiological cure as treatment goal without highlighting quality of life outcomes. This study assessed health-related quality of life (HRQoL) and related factors in the pre-treatment, intensive and continuation phases of anti-TB therapy among sputum smear positive pulmonary TB patients in Mbale region, Eastern Uganda.

**Methods:**

In this cross-sectional study, questionnaires and 36-Item Short-Form Health Survey Version 2.0 (UK English SF36v2) forms were administered to 210 participants of whom 64.8 % were males. The mean age was 35.48 ± 12.21 years. For each of the three treatment phases, different patients were studied. Responses were translated into the standard 00–100 scale. Means and standard deviations were used to express HRQoL as physical composite scores (PCS) and mental composite scores (MCS). Analysis of variance was used to compare scores across phases. Multiple linear regression methods were used to model relationships between predictor variables and HRQoL for each treatment phase.

**Results:**

HRQoL scores were different across treatment phases. General health (38.8 ± 17.5) and mental health (52.7 ± 18.6) had the lowest and highest sub-scale scores respectively. Mean PCS scores in pretreatment, intensive and continuation phases were 29.9 ± 19.4, 41.9 ± 14.2 and 62.2 ± 18.8 respectively. Mean MCS scores in the pretreatment, intensive and continuation phases were 38.8 ± 18.3, 49.4 ± 13.1 and 60.6 ± 18.8 respectively. Prior to treatment initiation, having an informal occupation (β = −28.66 (<0.001) was associated with poor HRQoL. Being unmarried (β = 11.94, p = 0.028) and belonging to the highest tertile of socioeconomic status (SES) (β = 14.56, p = 0.007) were associated with good HRQoL in the intensive phase. In the continuation phase, SES (β = 10.83, p = 0.021 for MCS and β = 13.14, p = 0.004 for PCS) predicted good HRQoL. Older age (β = −0.43 p = 0.013 for PCS and β = −0.36 p = 0.040 for MCS) was associated with poor HRQoL.

**Conclusions:**

TB treatment improved patients’ perceived health and having means of income was particularly associated with high HRQoL. Strategies to strengthen treatment support that include income generation and specific close monitoring of older patients may help improve overall TB treatment experience, by sustaining acceptable levels of physical, social and emotional functioning.

## Background

Tuberculosis (TB) drug therapy has been used as an effective strategy for prevention of mortality and morbidity. It involves a prolonged course of a combination of antibiotics that is divided into a 4-drug, 2-month intensive phase of rifampicin, isoniazid, pyrazinamide and ethambutol (2RHZE), followed by a continuation phase of two drugs administered for 4–6 months depending on the choice of regimen [3, 14, 17]. However, TB therapy presents some demerits that impact on patient’s health-related quality of life (HRQoL). This may be due to drug toxicity, adverse drug reactions [22], social stigma [10, 11] or the anxiety and a feeling of helplessness stimulated by the disease and its treatment processes [15]. These effects determine the way patients perceive their total health and wellbeing, which is defined as HRQoL [21]. HRQoL is a key aspect in the human health continuum because it affects treatment outcomes [7]. Much as HRQoL is now increasingly assessed in public health research and clinical practice as an appropriate indicator of intervention needs and outcomes in chronic diseases like TB, its incorporation into routine patient care and management protocols in Uganda is still limited.

A vital tool used to assess HRQoL at individual and population levels is the short form 36 version 2 (SF-36v2) which focuses on patients’ total wellbeing [20]. Related tools like medical outcomes survey form, are available and have been used in some studies in urban areas of Uganda where it was found that there are higher HRQoL scores for patients who had completed 8 months TB therapy compared to patients who had only just started their treatment [2]. The factors identified to influence health-related quality of life in tuberculosis include age, income, stigma, depression and family support [6, 12]. Although it is important to ascertain and monitor HRQoL of TB patients, there is still a narrow knowledge base on the subject in rural Uganda which is characterized by poor access to healthcare services. We therefore applied the SF36v2 form to TB patients in Mbale Regional Referral Hospital in order to examine their HRQoL and the possible predictors in order to generate information that may guide care and treatment.

## Methods

### Study setting and participants

This cross sectional study enrolled 210 smear positive pulmonary tuberculosis (PTB) patients receiving treatment from Mbale Regional Referral Hospital (MRRH) between March and July 2014. This hospital is situated in Eastern Uganda, on the foothills of Mountain Elgon, approximately 200 km from Kampala. MRRH has functional laboratories for confirmation of TB disease before treatment is initiated. Participants included inpatients and outpatients aged 18 years and above drawn from the pre-treatment phase as well as the intensive and continuation phases of TB therapy.

### Methods and materials

A sample size of 210 was calculated based on the formula provided by Friedman et al. for comparison of groups [9]. Seventy (70) patients in the pre-treatment phase were recruited consecutively when they presented for initial diagnosis. A stratified random sampling approach was applied to select 69 participants in the intensive phase and 71 patients in the continuation phase. A TB register was used to obtain a list of patients and dates on which treatment was started. Patients were then categorized according to treatment phase and numbers were assigned to them. Using the internet-based “http://www.random.org” engine, random numbers were generated and the first set of numbers totaling to sample size in each treatment phase were considered. Trained interviewers explained the study to eligible participants and obtained written consent before face to face interviews were conducted using the UK English SF36v2 form and a semi-structured questionnaire to collect data on HRQoL and associated factors respectively. Patients that were unable to communicate during the study for any reason like sickness were excluded.

The SF36v2 form and questionnaire were in both English and the local dialect, Lumasaaba. Participants were allowed to choose the language version of tools that they were most comfortable with. The participant’s response to each specific question was recorded on a Likert scale according to the SF-36v2 guidelines [20]. Data were collected on socio-demographic factors, family disclosure, family support, community awareness of patient’s TB status, community attitude towards the patient, and belonging to a social support group; clinical factors included HIV sero-status, sputum smear grade at initial diagnosis, self-reported alcohol consumption prior to diagnosis, TB treatment plan (community-based DOTS or facility-based DOTS); and economic factors were also collected. Information on the socio-economic status as measured by ownership of 11 indicator assets (radio, television, mobile phone, bicycle, motorcycle, motor vehicle, piece of land, large farm animals, small farm animals, manufactured bed, and the nature of the walls of their house) based on the Uganda Demographic and Health Survey was also captured.

### Data management and analysis

Responses recorded on the Likert scale were scored on a scale from 0 to 100, with 100 representing the highest level of functioning possible. Aggregate scores were then compiled as a percentage of the total points possible [19, 20]. Scores of responses to questions representing each specific area of functional health status were then averaged together, for a final score within each of the eight domains of HRQoL. For each domain, an aggregate percentage score was produced which ranged from 0 % (lowest level of functioning) to 100 % (highest level of functioning). The PCS was then computed as an average of the scale scores of self-perceived general health (GH), bodily pain (BP), physical functioning (PF) and physical role. MCS was calculated by averaging the scale scores of vitality (VT), social functioning (SF), mental health (MH) and emotional role (RE). Each of the final PCS and MCS scores was independently used to describe the HRQoL. To compare HRQoL, an analysis of variance (ANOVA) was performed at 95 % level of confidence to determine if at least one of the phases had a HRQoL score different from other phases.

Simple regressions were done to determine the independence of the covariates and a *p* value of 0.25 was considered as a cut off to determine which variables to carry over to the multiple linear regression analysis. Multiple linear regression analysis, with a backward elimination approach, was used to construct a parsimonious model for each HRQoL measure (i.e. PCS and MCS) in each treatment phase. Each model had factors that best explained the observed PCS or MCS scores in a given treatment phase. A total of six models were constructed i.e. two for each of the summary score in each of the three treatment phases. All statistical analyses were done using Stata 13 software (StataCorp, June 2013).

### Ethical considerations

Participants were given full and adequate oral and written information about the nature, purpose, possible risks and benefits of the study in both English and Lumasaaba languages. They were given adequate opportunity to ask questions and allowed time to consider the information provided. The participant’s signed and dated informed consent was obtained before conducting this study. The study data was stored in a computer database while maintaining confidentiality. Participants in this database were identified by the unique enrolment number only. Ethical clearance and permission was obtained from Makerere University School of Public Health Institutional Review Board, Mable Regional Referral Hospital and Mbale District Administration.

## Results

### Patient clinical and socio-demographic characteristics

A total of 210 patients were identified from the medical records and all accepted to participate in the study providing 100 % response level. Of these, 70 (33.3 %), 69 (32.9 %) and 71 (33.8 %) were in the pre-treatment, intensive and continuation phases of TB treatment, respectively. Patients in the intensive and continuation phases had spent mean durations of 31.90 (SD = 14.3, range = 3–60) and 132.3 (±48.2, range 61–237) days on treatment respectively. Most participants were males (64.8 %) and the mean age was 35.48 (SD = 12.21, range = 18–68) years. The mean age across the therapy phases did not differ (p = 0.624) and more than half of the patients (56.7 %) were married. About half (52.9 %) had either never received any formal education or had attained a maximum of primary education. Other characteristics are shown in Table 1.

**Table 1:** Characteristics of PTB sputum smear positive patients in Mbale regional referral hospital

Characteristic	Total population	Treatment phases			*X* ^*2*^ value (p value)
		Pretreatment	Intensive	Continuation	
	N = 210 (%)	N = 70	N = 69	N = 71	
Age (Mean ± SD)	35.48 ± 12.21	34.66 ± 12.05	36.61 ± 11.96	35.18 ± 12.70	0.472 (0.624)
Sex					
Male	136 (64.8)	47 (67.1)	46 (66.7)	43 (60.6)	0.832 (0.660)
Female	74 (35.2)	23 (32.9)	23 (33.3)	28 (39.4)	
Marital status					
Married/cohabiting	119 (56.7)	40 (57.1)	44 (63.8)	35 (49.3)	4.453 (0.348)
Separated	45 (21.4)	13 (18.6)	15 (21.7)	17 (23.9)	
Single/never married	46 (21.9)	17 (24.3)	10 (14.5)	19 (26.8)	
Family size^**^					
0–3	61 (29.0)	31 (44.3)	17 (24.6)	13 (18.3)	12.510 (0.002)
4+	149 (71.0)	39 (55.7)	52 (75.4)	58 (81.7)	
Education level					
None or primary	111 (52.9)	36 (51.4)	39 (56.5)	36 (50.7)	0.561 (0.755)
Secondary and above	99 (47.1)	34 (48.6)	30 (43.5)	35 (49.3)	
Religion^**^					
Christian	126 (60.0)	54 (77.1)	27 (39.1)	45 (63.4)	21.431 (≤ 0.001)
Non-christian	84 (40.0)	16 (22.9)	42 (60.9)	26 (36.6)	
HIV co-infection					
Yes	124 (59)	38 (54.3)	45 (65.2)	41 (57.7)	1.792 (0.408)
No	86 (41)	32 (45.7)	24 (34.8)	30 (42.3)	
Occupation					
Formal	50 (23.8)	13 (18.6)	22 (31.9)	15 (21.1)	3.820 (0.148)
Informal	160 (76.2)	57 (81.4)	47 (68.1)	56 (78.9)	
SES tertiles					
Lowest	84 (40.0)	26 (37.1)	26 (37.7)	32 (45.1)	4.407 (0.354)
Middle	87 (41.4)	29 (41.4)	34 (49.3)	24 (33.8)	
Highest	39 (18.6)	15 (21.4)	9 (13.0)	15 (21.1)	
Residence					
Typical rural/village	122 (58.1)	41 (51.6)	38 (55.1)	43 (60.6)	0.443 (0.801)
Trading center	88 (41.9)	29 (41.4)	31 (44.9)	28 (39.4)	
Initial sputum grade					
1+	48 (22.9)	16 (33.3)	15 (31.2)	17 (35.4)	0.459 (0.977)
2+	64 (30.5)	21 (32.8)	23 (35.9)	20 (31.2)	
3+	98 (46.7)	33 (33.7)	31 (31.6)	34 (34.7)	
Treatment strategy					
Community-based DOTS	33 (15.7)	10 (14.3)	14 (20.3)	9 (12.7)	1.693 (0.429)
Facility-based DOTS	177 (84.3)	60 (85.7)	55 (79.7)	62 (87.3)	
Community attitude					
Supportive	119 (56.7)	63 (90)	30 (43.5)	26 (36.6)	48.182 (≤ 0.001)
Not supportive	91 (43.3)	7 (10)	39 (56.5)	45 (63.4)	
Employment^**^					
Yes	112 (53.3)	53 (75.7)	34 (49.3)	25 (35.2)	23.913 (≤ 0.001)
No	98 (46.7)	17 (24.3)	35 (50.7)	46 (64.8)	
Disclosure to family^**^					
Yes	133 (63.3)	32 (45.7)	47 (68.1)	54 (76.1)	14.986 (0.001)
No	77 (36.7)	38 (54.3)	22 (31.9)	17 (23.9)	
Alcohol					
Yes	76 (36.2)	23 (32.9)	28 (40.6)	25 (35.2)	0.942 (0.624)
No	134 (63.8)	47 (67.1)	41 (59.4)	46 (64.8)	
Family support					
Yes	167 (79.5)	52 (74.3)	56 (81.2)	59 (83.1)	1.850 (0.397)
No	43 (20.5)	18 (25.7)	13 (18.8)	12 (16.9)	
Belonging to social support group					
Yes	27 (12.9)	5 (7.1)	9 (13.0)	13 (18.3)	3.926 (0.140)
No	183 (87.1)	65 (92.9)	60 (87.0)	58 (81.7)	

^**^Significant factors

### HRQoL domain scores in the pretreatment, intensive and continuation phases of treatment

There were significant differences in the mean scores for all HRQoL domains across treatment phases with the continuation phase having the highest. The physical health composite scores (PCS) were significantly different in the three treatment phases (p < 0.001) with the pretreatment phase having 29.9 ± 19.4, intensive phase 41.9 ± 14.2 and continuation phase possessing 62.2 ± 18.8. The overall PCS was 44.8 ± 22.1. The mental health composite scores (MCS) were significantly different in the three treatment phases (p < 0.001) with the pretreatment phase having 38.8 ± 18.3, intensive phase 49.4 ± 13.1 and continuation phase having 60.6 ± 18.8. The rest of the HRQoL subscale scores are shown in Table 2.

**Table 2:** HRQoL domains and summary scores across treatment phases among the 210 study participant

HRQoL domains	Treatment phases (N = 210)			
	Pretreatment	Intensive	Continuation	p value
	M (SD)	M (SD)	M (SD)	
Bodily pain (BP)	25.4 (23.9)	38.9 (23.1)	55.1 (28.9)	<0.001
Physical functioning (PF)	38.3 (27.4)	53.4 (23.3)	77.6 (20.2)	<0.001
General health (GH)	24.7 (13.9)	39.1 (11.3)	52.5 (14.3)	<0.001
Mental health (MH)	44.2 (18.7)	52.8 (15.8)	61.1 (17.5)	<0.001
Role limitations emotional (RE)	35.0 (30.7)	47.8 (24.2)	67.9 (30.3)	<0.001
Role limitations physical (RP)	31.2 (30.2)	36.2 (23.3)	63.7 (30.9)	<0.001
Social functioning (SF)	36.8 (27.6)	52.6 (20.9)	61.1 (30.2)	<0.001
Vitality (VT)	39.3 (15.5)	44.2 (13.4)	52.3 (14.0)	<0.001
Health transition (HT)	16.8 (30.0)	43.48 (31.1)	76.76 (22.9)	<0.001
HRQoL summary scores				
Physical health score (PCS)	29.9 (19.4)	41.9 (14.2)	62.2 (18.8)	<0.001
Mental health score (MCS)	38.8 (18.3)	49.4 (13.1)	60.6 (18.8)	<0.001

Table 2 shows that there is an increment in the PCS and MCS scores the longer a patient stays on treatment and PCS was greater than MCS in the continuation phase unlike in the pretreatment patients.

### Factors associated with health related quality of life

Across treatment phases, factors in the final adjusted regression models explained between 24.45 and 38.90 % of the observed variability in PCS. In the pretreatment phase, patients with formal employment were observed to have, on average, 30.27 % higher units in PCS scores compared to those with no employment (β = 30.27, p = <0.001). In the intensive and continuation phases, however, occupation did not affect PCS scores. In the intensive phase, patients whose marital status was single were observed to possess 11.94 % higher units in the PCS scores than those who were married. In the pretreatment and continuation phases, there was a significant average increment of 14.57 % units in the PCS score for patients in the highest tertile of socioeconomic status compared to those in the lowest tertile in the same phase. For every older year, a patient who was in the continuation phase was likely to have a reduction of 0.39 % units in the PCS score. However, age was not associated with PCS scores of the pretreatment and intensive phase. Other details are shown in Table 3.

**Table 3:** Factors associated with PCS Scores across treatment phases among sputum smear positive PTB patients in Mbale regional referral hospital

Factors	Pretreatment phase		Intensive phase		Continuation phase	
	Unadjusted β (p value)	Adjusted β (p value)	Unadjusted β (p value)	Adjusted β (p value)	Unadjusted β (p value)	Adjusted β (p value)
Age	−0.56 (0.003)	−0.13 (0.498)	−0.24 (0.101)	0.20 (0.203)	*−0.43 (0.013)*	*−0.43 (0.013)*
Sex						
Male	Ref		Ref		Ref	
Female	−4.06 (0.414)		3.58 (0.325)		−1.38 (0.765)	
Marital status						
Married	Ref	Ref	Ref	Ref	Ref	
Separated	−6.40 (0.276)	−3.23 (0.551)	−4.87 (0.213)	−6.61 (0.092)	−3.64 (0.516)	
Single	14.11 (0.009)	−1.93 (0.740)	*14.97 (0.002)*	*11.94 (0.028)*	4.26 (0.430)	
Family size						
0–3	Ref		Ref		Ref	
4+	7.15 (0.126)		4.01 (0.314)		3.93 (0.500)	
Education level						
None or primary	Ref		Ref		Ref	
Secondary and above	3.95 (0.397)		2.99 (0.387)		5.32 (0.236)	
Religion						
Christian	Ref		Ref		Ref	
Non-christian	6.19 (0.264)		−0.38 (0.914)		0.80 (0.864)	
HIV co-infection						
Yes	Ref	Ref	Ref	Ref		Ref
No	1.25 (0.790)	−3.68 (0.352)	2.35 (0.516)	−3.28 (0.307)	−1.66 (0.715)	−2.14 (0.623)
Alcohol consumption						
Yes	Ref	Ref	Ref		Ref	
No	−6.84 (0.167)	−2.42 (0.588)	−2.26 (0.520)		0.58 (0.902)	
Employment						
No	Ref	Ref	Ref		Ref	
Yes	−16.58 (0.002)	1.19 (0.855)	−4.28 (0.212)	−4.20 (0.191)	4.11 (0.382)	
Occupation						
Formal	Ref	Ref	Ref		Ref	
Informal	*−31.55 (<0.001)*	*−28.66 (<0.001)*	−6.52 (0.074)		−8.99 (0.100)	
SES tertiles						
Lowest	Ref		Ref	Ref	Ref	Ref
Middle	1.62 (0.758)		0.98 (0.783)	3.16 (0.342)	*13.11 (0.009)*	*12.78 (0.009)*
Highest	8.48 (0.181)		*14.36 (0.008)*	*14.56 (0.007)*	8.65 (0.131)	6.19 (0.248)
Residence						
Typical rural/village	Ref	Ref	Ref		Ref	
Trading center	10.06 (0.031)	4.24 (0.288)	−3.12 (0.366)		3.06 (0.507)	
Sputum grade						
1+	Ref		Ref	Ref	Ref	
2+	5.37 (0.411)		*9.31 (0.048)*	*13.26 (0.004)*	4.52 (0.467)	
3+	2.77 (0.643)		3.45 (0.434)	5.84 (0.152)	−3.71 (0.507)	
Treatment strategy						
Community-based DOTS	Ref		Ref		Ref	
Facility-based DOTS	0.0185 (0.998)		2.72 (0.526)		1.86 (0.784)	
Family disclosure						
No	Ref	Ref	Ref		Ref	
Yes	10.42 (0.024)	5.20 (0.222)	−0.60 (0.871)		4.60 (0.382)	
Family support						
Yes	Ref		Ref		Ref	
No	1.33 (0.805)		2.88 (0.513)		−5.06 (0.399)	
Community aware						
Yes	Ref		Ref		Ref	
No	26.49 (0.056)		3.31 (0.341)		2.11 (0.650)	
Community attitude						
Supportive	Ref	Ref	Ref		Ref	Ref
Not supportive	−2.21 (0.777)	−5.29 (0.443)	2.77 (0.425)		2.11 (0.650)	2.17 (0.623)
Social support group						
Yes	Ref		Ref		Ref	
No	1.74 (0.848)		−1.33 (0.794)		1.79 (0.759)	

In the pretreatment MCS adjusted regression model (Table 4), there was an increase of 25.15 % units in the MCS scores of the PTB patients with formal employment compared to those with no employment. Occupation however does not explain any of the observed MCS scores in the intensive and continuation phases. However, there was a reduction of 0.32 % units in the MCS amongst the continuation phase patients for every older year but age did not influence MCS scores of the pretreatment and intensive phases. There was also an increment of 10.83 % units in the MCS scores for continuation phase patients in the middle tertile of the SES compared to those in the lowest tertile. Other details of the MCS model are shown in Table 4.

**Table 4:** Factors associated with MCS Scores across treatment phases amongst sputum smear positive PTB patients in Mbale regional referral hospital

Factors	Pretreatment phase		Intensive phase		Continuation phase	
	Unadjusted β (p value)	Adjusted β (p value)	Unadjusted β (p value)	Adjusted β (p value)	Unadjusted β (p value)	Adjusted β (p value)
Age	−0.33 (0.072)	−0.14 (0.515)	−0.08 (0.536)	−0.03 (0.842)	*−0.36 (0.044)*	*−0.36 (0.040)*
Sex						
Male	Ref		Ref		Ref	
Female	−1.79 (0.704)		1.85 (0.584)		−1.97 (0.669)	
Marital status						
Married	Ref	Ref	Ref	Ref	Ref	
Separated	−3.50 (0.549)	−0.14 (0.981)	−0.44 (0.913)	−1.23 (0.767)	−3.99 (0.474)	
Single	6.46 (0.224)	−5.15 (0.399)	2.40 (0.606)	−0.76 (0.898)	5.60 (0.298)	
Family size						
0–3	Ref		Ref		Ref	
4+	4.96 (0.264)		−1.57 (0.670)		6.89 (0.235)	
Education level						
None or primary	Ref		Ref		Ref	
Secondary and above	4.10 (0.351)		−0.89 (0.781)		6.07 (0.176)	
Religion						
Christian	Ref	Ref	Ref		Ref	
Non-christian	6.20 (0.236)	6.26 (0.202)	−1.71 (0.600)		3.09 (0.509)	
HIV co-infection						
Yes	Ref		Ref		Ref	
No	−1.85 (0.677)		0.33 (0.919)		−1.66 (0.715)	
Alcohol consumption						
Yes	Ref		Ref	Ref	Ref	
No	−2.98 (0.525)		−3.93 (0.223)	−2.30 (0.521)	2.57 (0.586)	
Employment						
No	Ref		Ref	Ref	Ref	
Yes	−5.72 (0.264)		1.84 (0.562)	3.23 (0.355)	0.95 (0.840)	
Occupation						
Formal	Ref	Ref	Ref		Ref	
Informal	*−20.11 (<0.001)*	*−20.65 (0.001)*	−2.44 (0.474)		−4.71 (0.393)	
SES tertiles						
Lowest	Ref		Ref	Ref	Ref	Ref
Middle	3.25 (0.516)		−2.77 (0.414)	−3.30 (0.353)	*11.87 (0.019)*	*10.33 (0.038)*
Highest	4.51 (0.454)		6.57 (0.193)	6.70 (0.223)	7.96 (0.169)	8.54 (0.135)
Residence						
Typical rural/village	Ref		Ref		Ref	
Trading center	7.24 (0.102)		−1.29 (0.686)		3.06 (0.507)	
Sputum grade						
1+	Ref		Ref		Ref	
2+	5.35 (0.385)		3.47 (0.432)		5.24 (0.405)	
3+	2.63 (0.641)		2.98 (0.476)		1.17 (0.836)	
Treatment strategy						
Community-based DOTS	Ref		Ref		Ref	
Facility-based DOTS	6.96 (0.268)		4.69 (0.234)		6.09 (0.368)	
Family disclosure						
No	Ref	Ref	Ref	Ref	Ref	Ref
Yes	5.16 (0.242)	2.73 (0.524)	−3.99 (0.240)	−4.19 (0.237)	9.62 (0.065)	7.49 (0.175)
Family support						
Yes	Ref	Ref	Ref		Ref	
No	7.33 (0.143)	8.15 (0.083)	7.39 (0.066)		−4.89 (0.415)	
Community aware						
Yes	Ref		Ref		Ref	
No	11.40 (0.388)		0.55 (0.864)		3.37 (0.471)	
Community attitude						
Supportive	Ref		Ref	Ref	Ref	
Not supportive	−15.69 (0.030)		0.51 (0.874)	2.81 (0.415)	3.37 (0.471)	0.47 (0.922)
Social support group						
Yes	Ref		Ref	Ref	Ref	
No	9.21 (0.280)		−5.83 (0.215)	−8.08 (0.133)	−2.41 (0.679)	

## Discussion

Generally, HRQoL scores were higher in late phases of TB treatment and patients in the continuation phase had the best scores. PCS and MCS are highly correlated and there are several factors that are associated with PCS and MCS in different treatment phases.

There were significant differences in general health, bodily pain, physical functioning, physical role, vitality, social functioning, mental health and emotional role quality of life scores, and health transition in the three groups of PTB patients. Overall, the PCS and MCS scores were also significantly different in the three patient categories with the pretreatment patients having the lowest scores, followed by the intensive phase and those in the continuation phase having the highest scores. The finding of low HRQoL scores amongst pretreatment patients which are higher in later phases is in agreement with several studies [1, 2, 4, 13]. The low scores in the pretreatment patients may be explained by the negative feelings that are associated with the TB diagnosis and/or initial hospitalization [15] as a result of denial, stigma and self-blame. The HRQoL scores being higher in the continuation than intensive phase may probably be associated with a reduced pill burden and therapy benefits that the patients are drawing from the treatment, among other factors.

The HRQoL scores of this study are quite different from other studies of a similar nature. For example, the mental health score reported in Uganda [2] was 61.6 ± 25.5 whereas that of this study is 49.7 ± 19.1. The differences might have arisen as a result of the other study being exclusively urban and using a different HRQoL tool (MOS instrument) to evaluate the quality of life. A study in South Africa [16] found that TB patients had a mental health score of 55.6 ± 12.8. Another one in South Africa showed that pretreatment patients had PCS and MCS scores of 42.5 and 40.7 [12] respectively compared to this study’s 29.9 and 38.8 respectively on the SF-12 scale. These comparisons reveal that across different socio-cultural and economic contexts, the scale of measure (HRQoL tool) and TB disease impact the HRQoL of patients differently.

A strong positive correlation between PCS and MCS found in this study is in agreement with findings elsewhere that show direct proportionality between pain and depression [5]. People with bodily pain, which is characteristic of TB cases, often have worse mental and self-perceived health, than those without pain. This finding may mean that the mental and physical health components have separate but additive effects on an individual’s perceived well-being and that one can be used as a proxy for other.

During the pretreatment phase, being in formal employment was associated with high PCS and MCS probably because most of the formal jobs are done by educated individuals. Education may be playing an indirect role in this category of patients. Highly educated patients tend to have better health-seeking behavior and thus likely to report for treatment earlier than those who are less educated. They also integrate health-related information at a deeper level.

In the intensive phase, being single was positively associated with high PCS and not MCS. The positive association between being single and PCS may be explained by the lesser need to work hard and strain to meeting domestic/familial responsibilities amongst the participants who are not married. Higher socioeconomic status has been linked to treatment adherence and good treatment outcomes thus improved HRQoL scores [8, 12, 13] and our study was in agreement with such studies. This may probably be due to psychological effects associated with the treatment and reduced activity that makes the individual in lower SES redundant with reduced income. In such a state, low socioeconomic status individual tend to perceive their physical health in poor terms and perspectives.

In the continuation phase, age was consistently negatively associated with HRQoL in the PCS and MCS models. This finding is consistent with other studies [1]. In other treatment phases, age was not found to be associated with HRQoL scores just like in some other studies [12]. However, the finding of negative association between age and HRQoL supports the fact that health declines with aging because of the weakening of body systems. The observed positive correlation between SES and PCS is in agreement with findings of other studies [1, 13] may be due to the ability of those in higher SES to afford care while on treatment. For those in low SES, poverty aggravates social and health problems and the TB illness diminishes the motivation in the struggle to provide basic necessities thus the poor HRQoL scores.

Of importance to note is that unlike in many other studies [4, 12, 18], findings in this study show that family support, gender, alcohol and HIV co-infection were not significantly associated with HRQoL in any category of the patients that participated. This could have been due to homogeneity of the study participants. The findings on alcohol may have been due to the subjective nature of the questions asked on the subject. In this study, we did not measure the quantities of alcohol consumed daily or weekly hence this might have introduced reporting bias which may explain the negative finding on alcohol.

The study was cross-sectional in design which limits our analysis and conclusions on the cause-effect patterns. Furthermore, this study used the SF36v2 tool and designed questionnaire which were particularly quantitative. This tool was not validated in the study population and although this is likely to influence the outcome, the findings from this study were still consistent with studies in other population [1, 11]. Qualitative methods like in-depth interviews and focus group discussions would have provided deeper explanations behind the observed HRQoL scores.

## Conclusions

We conclude that HRQoL of smear positive PTB patients is lowest before treatment, indicating a decline in perceived health but it is higher in later phases with a peak in the continuation phase. We recommend that PTB patients are encouraged to undertake and adhere to anti-tuberculosis TB drug therapy and that economic activity should be encouraged amongst smear positive PTB patients. Additional special attention in terms of patient care, by healthcare providers and patient caretakers, should be paid to patients of older age in order to improve their perceived health.

## Abbreviations

AFB: acid-fast bacilli
ANOVA: analysis of variance
BP: bodily pain
CF: cognitive functioning
DTLC: district tuberculosis and leprosy control
HRQoL: health related quality of life
MCS: mental health component score
MH: mental Health
MRRH: Mbale Regional Referral Hospital
NTLP: National Tuberculosis and Leprosy Program
OR: odds ratio
PCS: physical health component summary
PF: physical functioning
PTB: pulmonary tuberculosis
RF: role functioning
SES: socioeconomic status
SF36v2: 36-Item Short-Form Health Survey Version 2.0
SD: standard deviation
SF: social functioning
TB: tuberculosis
UBOS: Uganda Bureau of Statistics
VT: vitality
WHO: World Health Organization

## References

[1] Atif M, Syed S, Syed S, Asrul A, Sarfraz M, Low M, Heng B, Zaheer-Ud-Din (2014). Impact of tuberculosis treatment on health-related quality of life of pulmonary tuberculosis patients: a follow-up study. Health Qual Life Outcomes.

[2] Babikako HM, Neuhauser D, Katamba A, Mupere E (2010). Feasibility, reliability and validity of health-related quality of life questionnaire among adult pulmonary tuberculosis patients in urban Uganda: cross-sectional study. Health Qual Life Outcomes.

[3] Centers for Disease Control and Prevention. Treatment for TB disease [Online]. Atlanta: Centers for Disease Control and Prevention; 2012. http://www.cdc.gov/tb/topic/treatment/tbdisease.htm Accessed 19 Feb 2014.

[4] Chamla D (2004). The assessment of patients’ health-related quality of life during tuberculosis in Wahan, China. Int J Turberc Lung Dis.

[5] Collingwood J. The relationship between mental and physical health. Psych Central. 2010. http://www.psychcentral.com/lib/the-relationship-between-mental-and-physical-health/0002949 Accessed 2 Sept 2014.

[6] Deribew A, Tesfaye M, Hailmichael Y, Negussu N, Daba S, Wogi A, Belachew T, Apers L, Colebunders R (2009). Tuberculosis and HIV co-infection: its impact on quality of life. Health Qual Life Outcomes.

[7] Dhingra VK, Rajpal S (2005). Health related quality of life (HRQL) scoring (DR-12 score) in tuberculosis—additional evaluative tool under DOTS. J Commun Dis.

[8] Duyan V, Kurt B, Aktas Z, Duyan GC, Kulkul DO (2005). Relationship between quality of life and characteristics of patients hospitalised with tuberculosis. Int J Tuberc Lung Dis.

[9] Friedman LM, Furberg CD, Demets DL (2010). Fundamentals of clinical trials.

[10] Kelly P (1999). Isolation and stigma: the experience of patients with active tuberculosis. J Commun Health Nurs.

[11] Kittikraisak W, Kingkaew P, Teerawattananon Y, Yothasamut J, Natesuwan S, Manosuthi W, Chongsuvivatwong V, Whitehead SJ (2012). Health related quality of life among patients with tuberculosis and HIV in Thailand. PLoS One.

[12] Louw J, Peltzer K, Naidoo P, Matseke G, Mchunu G, Tutshana B (2012). Quality of life among tuberculosis (TB), TB retreatment and/or TB-HIV co-infected primary public health care patients in three districts in South Africa. Health Qual Life Outcomes.

[13] Mamani M, Majzoobi MM, Ghahfarokhi SM, Esna-Ashari F, Keramat F (2014). Assessment of health-related quality of life among patients with tuberculosis in Hamadan, Western Iran. Oman Med J.

[14] Manabe YC, Hermans SM, Lamorde M, Castelnuovo B, Mullins CD, Kuznik A (2012). Rifampicin for continuation phase tuberculosis treatment in Uganda: a cost-effectiveness analysis. PLoS One.

[15] Marra CA, Marra F, Cox VC, Palepu A, Fitzgerald JM (2004). Factors influencing quality of life in patients with active tuberculosis. Health Qual Life Outcomes.

[16] McInerney PA, Nicholas PK, Wantland D, Corless IB, Ncama B, Bhengu B, McGibbon CA, Davis SM, Gallagher DM (2007). Characteristics of anti-tuberculosis medication adherence in South Africa. Appl Nurs Res.

[17] MOH (2010). Ministry of Health manual of the National Tuberculosis and Leprosy Programme.

[18] Peltzer K, Pengpid S (2012). Alcohol use and health-related quality of life among hospital outpatients in South Africa. Alcohol Alcohol.

[19] Ware JE, Kosinski M (2001). Interpreting SF-36 summary health measures: a response. Qual Life Res.

[20] Ware JE, Kosinski M, Dewey JE (2000). How to score version 2 of the SF-36 Health Survey.

[21] WHO. What quality of life? The WHOQOL Group. World Health Organization quality of life assessment, vol 17. Hague: World Health Forum; 1996. p. 354–6. 9060228

[22] Yee D, Valiquette C, Pelletier M, Parisien I, Rocher I, Menzies D (2003). Incidence of serious side effects from first-line antituberculosis drugs among patients treated for active tuberculosis. Am J Respir Crit Care Med.

